# Factors associated with self-efficacy in patients with hypertension: a cross-sectional study from Palestine

**DOI:** 10.1186/s41043-021-00225-2

**Published:** 2021-02-09

**Authors:** Salam Khairy, Asala Aslan, Ahmad M. Samara, Ibrahim Mousa, Abdulsalam S. Alkaiyat, Sa’ed H. Zyoud

**Affiliations:** 1 Department of Medicine, College of Medicine and Health Sciences, An-Najah National University, Nablus, 44839 Palestine; 2 Public Health Department, College of Medicine and Health Sciences, An-Najah National University Hospital, An-Najah National University, Nablus, 44839 Palestine; 3 Clinical Research Centre, An-Najah National University Hospital, Nablus, 44839 Palestine; 4 Department of Clinical and Community Pharmacy, College of Medicine and Health Sciences, An-Najah National University, Nablus, 44839 Palestine

**Keywords:** Hypertension, Self-Efficacy in the Management of Chronic Disease, Perceived Efficacy in Patient-Physician Interaction, Palestine

## Abstract

**Background:**

Self-efficacy is a widely applied psychosocial concept that is commonly used in association with management of chronic diseases, including hypertension. The aim of this study was to assess self-efficacy of hypertension management and patient-physician communication, as well as the factors associated with self-efficacy and patient-physician communication among patients with hypertension in Palestine.

**Methods:**

We conducted face-to-face, questionnaire-based interviews using validated instruments to assess self-efficacy in managing hypertension (Self-Efficacy for Managing Chronic Disease 6-Item Scale (SES6C)) and patient-physician communication (Perceived Efficacy in Patient-Physician Interaction Questionnaire (PEPPI)) in patients with hypertension at the three main primary healthcare centers in Nablus district in northern West Bank, Palestine. We also performed a multiple linear regression analysis to determine the variables independently associated with PEPPI and SES6C scores.

**Results:**

We enrolled 377 participants with hypertension in this study. The average age (measured in years) was 56.8 with a standard deviation of 11.6. The mean PEPPI and SES6C scores were 20.0 (SD 4.4) and 41.1 (SD 10.6), respectively. In a multiple linear regression model, subjects who were city dwellers (*B*=3.597, *p*=0.004), and subjects with high education levels (*B*=4.010, *p*=0.001) achieved higher PEPPI scores, whereas subjects in the normal weight category (*B*=5.566, *p*<0.001) and those with higher PEPPI scores (*B*=0.706, *p*<0.001) achieved higher SES6C scores.

**Conclusions:**

We found that impairment in self-efficacy was linked to overweight and obesity, as well as lower patient-physician communication. Moreover, our results showed that lower patient-physician communication was independently associated with low education level as well as non-city residency types. We recommend making the appropriate changes by both the policy-makers and the health care providers to improve the health facilities and its services, especially outside the cities. We also suggest holding specific counseling and training session on the management and control of hypertension.

## Background

Hypertension is considered a serious and frequent source of morbidity and mortality [1]. Moreover, it is one of the most common chronic illnesses in the world [2]. In fact, it is the number one cause of mortality and the third most common cause of disability-adjusted life years [3]. In Palestine, hypertension prevalence was estimated at 3.7% [4], and it was ranked the fifth leading cause of death, accounting for 8% of all deaths in 2016, at about 21.8 deaths per 100,000 population [5]. However, the rates of awareness, treatment, and control of hypertension were found to be low [6]**.** As an initial approach to hypertension management, patients are encouraged to make healthy changes in their lifestyle including more physical activity, weight reduction, smoking cessation, and stress control. They are also encouraged to follow a modified diet (salt restriction, potassium supplement, avoiding alcohol, and multi-factorial diet control).

In Palestine, the health-care providers have developed specific guidelines for hypertension management that comply with the international recommendations, while also take the Palestinian context in account [7]. Maintaining adherence to the medication used in treatment is considered as important as initiating the treatment [8]. Yet, a wide range research has found hypertension control to be poor and that preventive behaviors and management of risk factors are the main issues in treatment [9]. Therefore, many methods are needed for patients to strengthen the self-management of their treatment. Self-efficacy is a psychosocial concept that is commonly used in association with the ability to manage chronic diseases [6]. It was proved to play an important role in various areas including health-related behavior (such as relapse of smoking cessation), experience and management of pain, eating and weight control, successful recovery from myocardial infarction, and adherence to programs related to preventive health. Data also emphasize the important role of perceived self-efficacy as a cognitive factor that has an impact on health [6]. Several studies have investigated the pattern of self-efficacy and self-management behaviors in hypertensive patients worldwide [8–12].

Although several studies have been conducted in Palestine among hypertensive patients [13–19], none addressed self-efficacy among patients with hypertension. Therefore, there is no sufficient data to compare the characteristics of patients’ self-management in the Palestinian healthcare system to those of healthcare systems elsewhere. Cardiovascular disorders pose a major and increasing challenge to the health of the Palestinian population in Palestine [7, 20]. The proportions of awareness treatment and control of hypertension in Palestine were low [16]. The aim of this study was to assess self-efficacy of hypertension management and patient-physician communication, as well as the factors associated with self-efficacy and patient-physician communication among patients with hypertension in Palestine. The rationale behind this assessment is to provide the bases for the efforts aimed at giving patients the necessary tools to achieve self-efficacy, developing educational sessions to improve self-efficacy practices among patients with hypertension, and implementing thorough programs that engage the families of these patients, which will ultimately improve treatment outcomes.

## Methods

### Study design and setting

For the current survey, we used the cross-sectional design. The study took place at three primary healthcare centers in Nablus city in northern West Bank in Palestine, which are the main healthcare providers that serve the general population of Nablus district including Nablus city and the villages and camps of the region, including the hypertensive patients. Data collection was completed between July and December 2017.

### Sample size and sampling technique

We used Raosoft, an automated sample size calculating software (http://www.raosoft.com/samplesize.html). A minimum effective sample of 377 was calculated using to the following parameters: first, the estimated number of hypertensive patients in Nablus district (the target population) was 27000 [5]; second, the response distribution for the different questions of the survey was assumed at 50%; third, the margin of error was taken as 5% as the maximum amount of error that can be tolerated; and last, the confidence level was set at 95% following what is most often used in biomedical research [21]. The minimum effective sample was determined only to ensure that we included the minimum number of participants required to justify the results of the analysis. Participants were recruited by convenience sampling. The purpose of the sampling method was to provide an equivalent sample size from each primary health center wherein a sample of around 133 hypertensive patients was selected from each primary healthcare centre. During the study period, investigators visited the healthcare centres on a regular basis to recruit and interview potential participants. The visits were made in the morning time because it is the assigned time for hypertension clinics to provide care for patients with hypertension.

### Inclusion and exclusion criteria

All participants included in this survey met the following criteria: age of at least 18 years, the ability to read or comprehend Arabic, having been the diagnosis of hypertension for 6 months or more, and being medically treated for hypertension at the time of the study. Hypertension was defined as a mean systolic blood pressure (SBP) ≥ 130 mm Hg, mean diastolic blood pressure (DBP) ≥ 80 mm Hg, and/or use of antihypertensive medications. Patients diagnosed with mental illness or significant cerebrovascular disease were excluded from this study due to the possible effect of such diseases on their cognitive abilities.

### Questionnaire

In this study, a four-section questionnaire was used to collect the data.

- The first section inquired about the sociodemographic characteristics including age, sex, type of residency, employment, their primary health care center, marital status, the level of education, body mass index (BMI) category (normal if BMI was between 18 and 24.9, overweight if BMI was between 25 and 30, and obese if BMI was more than 30), and monthly income.
- The second section contained items on hypertension and related risk factors, such as the smoking status, the duration of the hypertension history, and co-morbid illnesses, as well as medications for hypertension treatment, their dosages, and for how long they have been on each treatment.
- The third section addressed perceived self-efficacy using the Perceived Efficacy in Patient-Physician Interaction Questionnaire (PEPPI). This tool focuses on obtaining medically relevant data as well as concerns of the patients from the physician. Subjects select a number between 1 and 5 to respond to each question with 1 meaning they have no confidence at all, whereas 5 meaning there have high confidence, with the total result ranging from 5 to 25 where the higher the score the higher self-efficacy in patient-physician interactions is [22, 23]. The PEPPI as used in the current survey showed evidence for reliable internal consistency as indicated by Cronbach’s alpha coefficient of 0.871.
- The fourth section employed Self-Efficacy for Managing Chronic Disease 6-Item Scale (SES6C) which can be used on a variety of chronic diseases. It contains a 6-item scale focusing on deferent areas in order to measure the level of confidence among the participants in these areas. The areas of focus are controlling symptoms, functioning role, emotional function, and communicating with the physician. A 10-point rating scale, in which 1 stands for not confident at all and 10 stands for totally confident, is used to calculate the score for each of these 6 questions. The scores of these scales are then added up with the sum falling in the range of 6 to 60, where a higher score means better perceived self-efficacy for managing chronic illnesses [24]. The SES6C in our study showed evidence of reliable internal consistency as indicated by Cronbach’s alpha coefficient of 0.934. The Arabic version of the questionnaire including the translated PEPPI and SES6C was tested on a pilot sample of 30 patients before the official data collection began. The pilot results were excluded from the main survey analyses and results.

### Ethical issues

The current survey received approval from the Institutional Review Board (IRB) at An-Najah National University (Protocol Reference No.: 3SEP2017). Appropriate written approvals were provided by the Palestinian ministry of health in order to collect data from all included primary health care centers. Additionally, the participants were asked to provide a verbal consent to be included in the study.

### Data analysis

Statistical analysis was conducted by version 21 of IBM SPSS Statistics (IBM Corp. Released 2012. IBM SPSS Statistics for Windows, Version 21.0. Armonk, NY: IBM Corp). Descriptive analyses were employed in calculating the means, medians, standard deviations, and inter-quartiles values for variables of the study. The normality distribution of continuous data was analysed by using the Kolmogorov–Smirnov test. Mann–Whitney *U* test and Kruskal–Wallis test were both utilized to compare self-efficacy and PEPPI scores between different participants’ categories according to their characteristics. Pearson correlation was performed to establish relationships between PEPPI and SES6C scores. Any independent variable with a *p* value of < 0.05 was considered a candidate for multiple linear regression. Multiple linear regression analyses were carried out for the identification of determinants (categorical and continuous independent variables) associated with PEPPI and SES6C scores (continuous dependent variables). Multiple regression is used to analyze the relationship between a continuous dependent variable and a number of independent variables or predictors (usually continuous) and may also be used by dichotomous independent variables [25]. A dummy coding of 0 and 1 was used to enter the nominal independent variables into the regression model [26]. Colinearity between independent variables was assessed by calculation of the variance inflation factor (VIF). Multicollinearity in multivariable regressions was considered as not problematic if VIF of the variables was < 2. The level of statistical significance was assumed at *p*<0.05.

## Results

### Demographic and clinical characteristics

A total of 400 questionnaires were collected. After excluding 23 questionnaires for missing data and other exclusion criteria, the final analysis was conducted on 377 hypertensive patients. Table 1 presents the demographic and clinical characteristics of the participants in detail. The participants’ mean age was 56.8 (in year), and the standard deviation was 11.6. The majority (73.2%) belonged to the age group of less than a 65-year old. According to their residency, 70.8% were city residents. The sample had almost equal numbers of male and female participants. Only a minority (14.6%) of the subjects had BMIs within the normal range, while the majority were either overweight (32.4%) or obese (53.1%). Over three-quarters of the participants had received at least high school level of education and slightly over half of them were unemployed. The majority of the patients have been diagnosed with hypertension for a minimum of 5 years (75.6%) and were treated with a single antihypertensive drug (66.8%). Only 19.4% of them had more than one other chronic disease (Table 1).

**Table 1:** Demographic characteristics of participants

Variable	Number (%)
**Gender**	
Male	194 (51.5)
Female	183 (48.5)
**Age (year)**	
Less than 65	276 (73.2)
65 or more	101 (26.8)
**Residency**	
Village	76 (20.2)
City	267 (70.8)
Refugee camp	34 (9.0)
**Employment**	
Employed	170 (45.1)
Unemployed	207 (54.9)
**BMI**	
Normal	55 (14.6)
Overweight	122 (32.4)
Obese	200 (53.1)
**Marital status**	
Married	312 (82.8)
Unmarried	65 (17.2)
**Monthly income (NIS)** ^**a**^	
Low (less than 2000)	98 (26.0)
Moderate (2000–5000)	227 (60.2)
High ( more than 5000)	52 (13.8)
**Education level**	
Uneducated	16 (4.2)
Primary school	69 (18.3)
High school	136 (36.1)
University	156 (41.4)
**Hypertension duration**	
Less than 1 year	13 (3.4)
1–3 years	58 (15.4)
4–5 years	21 (5.6)
5 years or more	285 (75.6)
**Therapy type**	
Monotherapy	252 (66.8)
Multi therapy	125 (33.2)
**Total number of chronic diseases** ^b^	
1 or less	304 (80.6)
2	67 (17.8)
3 or more	6 (1.6)
**Total number of medications**	
Less than 4	273 (72.4)
4 or more	104 (27.6)

*NIS* New Israeli Shekel

^a^1 Israeli New Shekel equals 0.29 US dollar

^b^Total number of chronic diseases other than hypertension

### Relationship between characteristics of patients and self-efficacy score

The average SES6C score of the subjects was 41.1 (standard deviation = 10.6) out of a maximum score of 60. Many characteristics were associated with higher SES6C score as shown in Table 2. Patients less than 65 years old had a higher SES6C score compared to those aged 65 years or older (*p* value = 0.019). Also, a higher score was found among patients with normal BMI compared to overweight and obese patients (*p* value < 0.001). Residing in a city, being employed, and having a higher educational level were all associated with a better SES6C score (*p* values <0.001 for all). Other characteristics that were not associated with a statistically significant difference in SES6C score were gender, monthly income, hypertension duration, time of therapy, other chronic diseases’ number, and the number of medication the patient is taking in total.

**Table 2:** Relationship between characteristics of patients and self-efficacy score

Variable	Median [Q1–3]	***P*** value^**a**^
**Gender**		
Male	42.0 [36.0–48.0]	0.319^c^
Female	41.0 [32.0–48.0]	
**Age (year)**		
Less than 65	42.0 [36.0–48.0]	**0.019**^c^
65 or more	36.0 [27.5–51.0]	
**Residency**		
City	42.0 [36.0–50.0]	**<0.001**^c^
Village or refugee camp	36.0 [30.0–46.0]	
**Employment**		
Employed	42.0 [38.0–50.25]	**<0.001**^c^
Unemployed	39.0 [31.0–38.0]	
**BMI**		
Normal weight	48.0 [41.0–54.0]	**<0.001**^c^
Overweight or obese	41.0 [33.0–48.0]	
**Marital status**		
Married	42.0 [35.25–48.0]	0.104^c^
Unmarried	39.0 [29.5–52.5]	
**Monthly income (NIS)**^**b**^		
Low (less than 2000)	39.0 [30.0–48.0]	0.056^c^
Moderate to high income (≥2000)	42.0 [36.0–48.0]	
**Education level**		
Below university education level	39.0 [32.0–47.5]	**<0.001**^**c**^
University education level	43.0 [38.0–52.0]	
**Hypertension duration**		
Less than 1 year	42.0 [31.5–51.0]	0.897^d^
1–3 years	42.0 [35.25–50.0]	
4–5 years	42.0 [36.0–51.5]	
5 years or more	42.0 [33.5–48.0]	
**Therapy type**		
Monotherapy	42.0 [34.25–49.0]	0.102^c^
Multi-therapy	41.0 [32.0–48.0]	
**Total number of chronic diseases**		
1 or less	42.0 [34.0–48.0]	0.095^d^
2	42.0 [33.0–48.0]	
3 or more	23.0 [23.0–40.5]	
**Total number of medications**		
Less than 4	42.0 [33.0–49.0]	0.594^c^
4 or more	41.0 [35.0–47.0]	

Self-Efficacy for Managing Chronic Disease 6-Item Scale (SES6C) is range between 6 and 60, in which the higher the score, the higher the perceived self-efficacy for managing chronic diseases

*NIS* New Israeli Shekel; Q1–Q2, lower quartile and the upper quartile

^a^*P* values are bold where they are less than the significance level of 0.05

^b^1 Israeli New Shekel equals 0.29 US dollar

^c^Statistical significance of the differences determined by the Mann-Whitney *U* test

^d^Statistical significance of the differences determined by the Kruskal-Wallis test

### Relationship between characteristics of patients and PEPPI score

The average PEPPI score of the subjects was 20 points out of the maximum 25-point score (standard deviation = 4.4). Table 3 demonstrates the association between characteristics of patients and PEPPI score. Gender, residency, marital status, monthly income, and education level were associated with significant difference in PEPPI score. Male gender and being married were both associated with a higher score, (*p* values were 0.035 and 0.032, respectively). Also, city residents, those with a higher level of education and those with a higher monthly income had achieved higher scores (*p* values were <0.001 for monthly income and educational level and 0.010 for residency). The rest of the characteristics (age, employment, BMI, hypertension duration, therapy type, number of other chronic diseases, and the number of medications the patients took in total) did not have any significant impact on the participants’ PEPPI score.

**Table 3:** Relationship between characteristics of patients and PEPPI score

Variable	Median [Q1–Q3]	***P*** value^**a**^
**Gender**		
Male	20.0 [18.0–25.0]	**0.035**^**c**^
Female	20.0 [17.0–23.0]	
**Age (year)**		
Less than 65	20.0 [17.0–23.0]	0.846^c^
65 or more	20.0 [16.0–25.0]	
**Residency**		
City	21.0 [18.0–24.0]	**<0.010**^c^
Village or refugee camp	19.0 [15.0–23.0]	
**Employment**		
Employed	20.0 [18.0–24.2]	0.054^c^
Unemployed	20.0 [16.0–23.0]	
**BMI**		
Normal weight	22.0 [17.0–24.0]	0.682^c^
Overweight or obese	20.0 [17.0–24.0]	
**Marital status**		
Married	20.0 [17.3–24.7]	**0.032**^c^
Unmarried	19.0 [15.0–23.0]	
**Monthly income (NIS)**^**b**^		
Low (less than 2000)	18.0 [15.0–23.0]	**<0.001**^c^
Moderate to high income (≥2000)	20.0 [18.0–24.0]	
**Education level**		
Below university education level	20.0 [17.0–23.0]	**<0.001**^c^
University education level	22.0 [18.3–25.0]	
**Hypertension duration**		
Less than 1 year	20.0 [18.5–22.5]	0.980^d^
1–3 years	20.0 [17.0–23.3]	
4–5 years	20.0 [16.5–23.5]	
5 years or more	20.0 [17.0–24.0]	
**Therapy type**		
Monotherapy	20.0 [18.0–24.0]	0.456^c^
Multi therapy	20.0 [16.0–24.0]	
**Total number of chronic diseases**		
1 or less	20.0 [17.0–23.0]	0.402^d^
2	21.0 [17.0–25.0]	
3 or more	13.0 [11.0–27.5]	
**Total number of medications**		
Less than 4	20.0 [17.0–23.5]	0.765^c^
4 or more	20.0 [17.0–24.0]	

Perceived Efficacy in Patient-Physician Interaction Questionnaire (PEPPI) is range of 5 to 25; higher scores indicate that the participant has higher self-efficacy in patient-physician interactions

*NIS* New Israeli Shekel; Q1–Q2, lower quartile and the upper quartile

^a^*P* values are bold where they are less than the significance level of 0.05

^b^1 Israeli New Shekel equals 0.29 US dollar

^c^Statistical significance of the differences determined by the Mann-Whitney *U* test

^d^Statistical significance of the differences determined by the Kruskal-Wallis test

### Correlations between PEPPI score and SES6C score

The mean PEPPI and SES6C scores were 20.0 (SD 4.4) and 41.1 (SD 10.6), respectively. The two scores were positively correlated with each other (*r*=0.343; *p*<0.001).

### Factors associated with PEPPI score

Multiple linear regression analysis was performed for the identification of the determinants associated with PEPPI score (Table 4). The independent variables were the factors significantly associated with PEPPI score (the dependent variable in this case) in the univariate analyses including gender, residency, marital status, monthly income, and education level (Table 3). In a multiple linear regression regression model, subjects with hypertension, particularly city dwellers (*B*=3.597, *p*=0.004), and those who had higher level of education (*B*=4.010, *p*=0.001) had a positive association with PEPPI score. The VIFs for all independent variables were close to one (range 1.068–1.319), which indicates that there was no collinearity.

**Table 4:** Multivariable linear regression analysis showing independent variables associated with the PEPPI score in hypertension patients

Variables^**a, b**^	Unstandardized coefficients		Standardized coefficients	***t***	***P*** value^**c**^	95% confidence interval for ***B***
	***B***	Std. Error	Beta			
**Gender** Female Male	0.097 Ref.	1.130	0.005	0.086	0.931	− 2.125–2.320
**Residency** City Village or refugee camp	3.597 Ref.	1.246	0.154	2.885	**0.004**	1.145–6.045
**Marital status** Married Unmarried	1.734 Ref.	1.444	0.062	1.201	0.230	− 1.105–4.573
**Monthly income** ^c^ Moderate to high income Low income	− 0.260 Ref.	1.381	− 0.011	− 0.188	0.851	− 2.976–2.456
**Education level** ^c^ University education level Below university education level	4.010 Ref.	1.163	0.187	3.448	**0.001**	1.723–6.298

Perceived Efficacy in Patient-Physician Interaction Questionnaire (PEPPI) is range of 5 to 25; higher scores indicate that the participant has higher self-efficacy in patient-physician interactions

^a^Univariate variables with *P* values < 0.05 were entered into the multiple linear regression

^b^Nominal variables were entered into analysis using dummy coding

^c^*P* values are bold where they are less than the significance level of 0.05

### Factors associated with SES6C scores

Multiple linear regression analysis was performed for the identification of the determinants associated with SES6C scores (Table 5). The independent variables were the factors significantly associated with SES6C scores (the dependent variable in this case) in the univariate analyses including age, residency, employment, BMI, education level, and PEPPI Score (Table 2). In a multiple linear regression regression model, subjects with hypertension, particularly the ones in the normal weight category (*B*=5.566, *p*<0.001), and those with higher PEPPI scores (*B*=0.706, *p*<0.001) had a positive association with SES6C scores. The VIFs for all independent variables were close to one (range 1.057–1.277), which indicates that there was no collinearity.

**Table 5:** Multivariable linear regression analysis showing independent variables associated with the self-efficacy score in hypertension patients

Variables^**a, b**^	Unstandardized coefficients		Standardized coefficients	***t***	***P*** value^**c**^	95% confidence interval for ***B***
	***B***	Std. Error	Beta			
**Age category (years)** <65 ≥65	1.766 Ref.	1.164	0.074	1.518	0.130	− 0.522–4.054
**Residency** City Village or refugee camp	2.111 Ref.	1.131	0.091	1.866	0.063	− 0.114–4.336
**Employment** Employed Unemployed	1.960 Ref.	1.107	0.092	1.770	0.078	− 0.217–4.137
**BMI** ^c^ Normal weight Overweight or obese	5.566 Ref.	1.420	0.186	3.920	**< 0.001**	2.774–8.358
**Education level** ^c^ University education level Below university education level	1.932 Ref.	1.101	0.090	1.754	0.080	− 0.233–4.098
**PEPPI Score (continuous)**	0.706	0.114	0.294	6.195	**< 0.001**	0.482–0.930

Self-Efficacy for Managing Chronic Disease 6-Item Scale (SES6C) is range between 6 and 60, in which the higher the score, the higher the perceived self-efficacy for managing chronic diseases

^a^Univariate variables with *P* values < 0.05 were entered into the multiple linear regression

^b^Nominal variables were entered into analysis using dummy coding

^c^*P* values are bold where they are less than the significance level of 0.05

## Discussion

In this study, we evaluated the self-efficacy and patient-physician communication among Palestinian hypertensive patients and explored the association between deferent demographic and clinical characteristics in one hand and the self-efficacy (using the SES6C) and the patient-physician interaction (using the PEPPI scale) in the other hand. We found that hypertensive patients, in general, had a good self-efficacy and PEPPI scores. We also found that gender, type of residency, employment state, BMI, and educational level were all associated with a significant difference in the SES, while only residency and educational level had a significant impact on the PEPPI score among hypertension patients.

Male gender and city residency were associated with a higher self-efficacy scale. This finding is in discordance with the findings of a Chinese study conducted in 2015 [27] which found no significant association of self-efficacy with sex, although their findings in relation to age, marital status, and hypertension duration in patients were similar to our findings, where there was no significant correlation between these variables and patients’ self-efficacy.

Patients with normal BMI achieved a higher score regarding self-efficacy. This is similar to the results found in a study conducted on African-American hypertension patients where a significant association between good self-efficacy and good weight management strategies was reported [6]. This association can also be attributed to the fact that people with higher BMI, in general, tend to be less physically active, which in turn can negatively affect their self-efficacy as was found in a previous study conducted in Nigeria [28]. This association between self-efficacy and physical exercise has also been reported in many studies including a study conducted in china [11] and a more recent study conducted in Pakistan [29].

We also found a significantly higher self-efficacy score among both employed patients and those with higher educational level compared to unemployed and less educated patients, respectively. These results are in consistency with those of previous studies where similar associations were reported [27, 29].

As for the perceived efficacy in patient-physician interaction, a positive correlation between PEPPI score and patients’ level of educational was found in this study. This finding is in consistency with that of a similar study conducted in the USA [23].

Also, patients’ residency had a significant impact on their PEPPI score. This was evident from the higher PEPPI score achieved by city resident compared to those residing outside the city.

### Strengths and limitations

The main strength in the current survey was its originality, as it was the first study to assess self-efficacy and patient-physician interaction in hypertension patients as well as to apply the SES and PEPPI in Palestine. Moreover, the data in this study was collected directly from the patients, which may have better reflected the actual situation of those patients. Other strengths include the multi-centric setting and the relatively large sample size. On the other hand, there are certain limitations to this study. The first is related to the nature of the sample that utilized a convenience sampling technique. Secondly, because the design of this study was cross-sectional, it was not possible to assess the temporal relationships between self-efficacy and other hypertension-related variables.

## Conclusions

The current study discussed factors that impair self-efficacy and patient-physician interaction among patients with hypertension. We found an association between the level of impairment in self-efficacy and overweight and obesity and low patient-physician communication. Moreover, our results showed that lower patient-physician communication was independently associated with low level of education as well as non-city residency. We recommend making the appropriate changes by the policy-makers and the health-care providers to improve the health facilities and their services, especially outside the main cities. We also suggest holding specific counseling and training sessions on the topic of management and control of hypertension. On the other side, patients should be encouraged to make positive life-style modifications, especially in regard to physical exercises, which can be achieved through targeted health promotion and counseling programs. Additionally, this assessment provides the basis for the health-care providers to increase skills, knowledge, motivation, self-esteem, and awareness regarding self-efficacy among patients on medical therapy for hypertension through developing intervention plans and empowering systems that already focus on this issue.

## Data Availability

The datasets used and/or analyzed during the current study are available from the corresponding author on reasonable request.

## Abbreviations

SES6C: Self-Efficacy for Managing Chronic Disease 6-Item Scale
PEPPI: Perceived Efficacy in Patient-Physician Interaction Questionnaire
VIF: Variance inflation factor
IRB: Institutional Review Board
SD: Standard deviation
BMI: Body mass index
NIS: New Israeli Shekel
ESRD: End-stage renal disease
DM: Diabetes mellitus
BMI: Body mass index
JD: Jordanian dinner
MOH: Ministry of Health
